# Patient-derived cells – an irreplaceable tool for research of reduced penetrance in movement disorders

**DOI:** 10.1515/medgen-2022-2133

**Published:** 2022-08-12

**Authors:** Philip Seibler, Aleksandar Rakovic

**Affiliations:** Institute of Neurogenetics, University of Lübeck, Lübeck, Germany

**Keywords:** reduced penetrance, genetic modifiers, endogenous human models, iPSC, organoid

## Abstract

Movement disorders comprise a clinically, pathologically, and genetically heterogeneous group of diseases associated with the phenomenon of reduced penetrance. Penetrance refers to the likelihood that a clinical condition will occur when a particular genotype is present. Elucidating the cause of reduced penetrance may contribute to more personalized medicine by identifying genetic factors that may prevent individuals from developing disease. Therefore, patient material becomes an irreplaceable resource in this approach. It is needed to identify genetic modifiers of the disease in the first place and to subsequently elucidate underlying mechanisms in endogenous human cell models that provide the entire genetic background.

## Introduction

Movement disorders (ataxia, dystonic disorders, gait disorders, Huntington disease, myoclonus, parkinsonism, spasticity, tardive dyskinesia, tics and tremor) are clinically, pathologically and genetically heterogeneous and are characterized by impairment of the planning, control or execution of movement. The Movement Disorder Society Genetic mutation database (https://www.mdsgene.org) provides a comprehensive overview of published data on movement disorder patients reported to carry gene mutations. However, the distinction between disease-causing and risk-conferring is not clear in many instances. In addition, the pathological impact of a mutation may vary significantly between different individuals.

Reduced penetrance and variable expressivity are factors that influence the effects of particular genetic changes. A condition is said to show reduced penetrance, when some individuals who carry the pathogenic variant express the associated trait while others do not. Reduced penetrance is therefore a phenomenon well documented in hereditary diseases [1]. In conjunction with disease traits, it needs to be considered that penetrance is usually age-dependent and may border on the extent to which the phenotype is expressed in cases of late or subtle disease manifestations. For example, the penetrance of mutations in *beta-glucocerebrosidase* (*GBA*), the most frequent risk factor for Parkinson disease (PD), was estimated as 7.6 %, 13.7 %, 21.4 %, and 29.7 % at 50, 60, 70, and 80 years, respectively [2].

Elucidating the cause of reduced penetrance may contribute to a more personalized medicine even in the absence of a complete understanding of the disease mechanism itself. The combination of next-generation sequencing technologies and high-throughput genotyping platforms have provided invaluable insight not only into risk variants of disease but also into genetic factors that may prevent individuals from developing disease [3]. This concept of modifier genes offers the possibility to specifically test for modifier genes and protective alleles among at-risk individuals and to study the efficacy of therapeutics based on the genetic background of individuals.

Therefore, patient material becomes an irreplaceable resource in this approach. It is needed to identify genetic modifiers of the disease and to subsequently elucidate underlying mechanisms. Only patient-derived cells provide the entire genetic background and carry all genetic alterations, including epigenetic modifications, that enable such studies.

## Genetic modifiers and penetrance biomarkers in Parkinson disease

PD is recognized as the most common neurodegenerative movement disorder and is characterized by the clinical features bradykinesia, rigidity, rest tremor, and postural instability. It is defined at post-mortem by the loss of dopaminergic neurons in the substantia nigra pars compacta, typically accompanied by the presence of protein aggregates called Lewy bodies. Despite the increasing understanding of PD, there is no neuroprotective cure for PD, only treatments that provide symptomatic relief.

PD is generally considered a multifactorial disorder that arises owing to a combination of genetic and environmental factors. Among these, approximately 5 % of the idiopathic cases can currently be identified as PD syndromes caused by a single genetic event [4]. Over the past two decades, genes have clearly been linked to monogenic PD, including three autosomal dominantly inherited ones (*alpha-synuclein* (*SNCA*), *leucine-rich repeat kinase 2* (*LRRK2*), and *vacuolar protein sorting-associated protein 35* (*VPS35*)) and three recessively transmitted ones (*E3 ubiquitin ligase Parkin* (*Parkin*), *PTEN-induced putative kinase 1* (*PINK1*), and *protein deglycase DJ-1* (*DJ-1*)). In addition, variants at the SNCA locus and heterozygous mutations in *GBA* have been discovered as significant genetic risk factors for idiopathic PD. Over the past decade, collaborative groups have worked together to further investigate the genetic basis of idiopathic PD. In a recent meta-analysis, datasets from genome-wide association studies (GWAS) available for PD have been examined to identify novel loci for disease risk and 90 independent genome-wide significant risk signals across 78 genomic regions were found [5]. These 90 variants explained 16–36 % of the heritable risk of PD depending on prevalence. The study also provided a biological context for these risk factors and showed that a considerable genetic component of PD remains unidentified.

Growing evidence has suggested a close correlation between genetic variation and age at onset (AAO) of PD. For example, several Mendelian genes with recessive inheritance patterns, such as *Parkin*, *PINK1*, and *DJ-1*, cause early-onset PD, while mutations in *LRRK2* are more common in late-onset PD. Interestingly, some PD risk loci have been established to be associated with AAO [6], [7], [8], [9]. For LRRK2 p.G2019S parkinsonism, genetic variability within *DNM3* has been found as an AAO modifier of disease [7]. In search of a penetrance biomarker for LRRK2-associated PD, it was found that nonmanifesting LRRK2 mutation carriers had significantly higher levels of urate, an antioxidant, transcription factor Nrf2 activator, and inverse risk factor for idiopathic PD, than those who developed PD [10]. Moreover, a link between mtDNA integrity and disease progression has been investigated showing that levels of somatic mtDNA deletions in LRRK2 mutation carriers were associated with PD status [11]. In accordance with that, affected p.G2019S carriers showed reduced NADH dehydrogenase activity, elevated mitochondrial mass and mtDNA copy numbers as well as increased expression of Nrf2 [12]. The role of heterozygous mutations in *Parkin* and *PINK1* as a risk factor for PD is still under debate as epidemiological analyses provided controversial results [4]. However, there is evidence that heterozygous mutations increase the risk of developing PD with an AAO falling between that of biallelic mutation carriers and idiopathic PD patients [13], [14], [15]. Molecular studies of these modifier genes in patient cells provide unique opportunities to investigate the mechanisms of neuronal degeneration in endogenous human models of PD.

## Genetic modifiers in dystonia

Dystonia is a movement disorder characterized by sustained or intermittent muscle contractions causing abnormal, often repetitive movements and/or postures. Forms of dystonia without neurodegeneration usually reach a plateau with a stable phenotype, whereas those associated with neuronal loss progressively worsen over time. It is a heterogeneous group of syndromes and in the past three decades, more than 200 genes have been linked to different, mostly childhood-onset generalized forms of dystonia [16].

Mutations in *TOR1A* are the most common inherited form of dystonia with still unclear pathophysiology and reduced penetrance of 30–40 %. The p.D216H TOR1A polymorphism has been described as genetic modifier [17]. Analysis of haplotypes demonstrated a highly protective effect of the H allele in trans with the frequent 3-bp deletion in the *TOR1A* gene (c.907_909delGAG) and there was also suggestive evidence that the D216 allele in cis is required for the disease to be penetrant. However, this observation explains only a small proportion of the reduced penetrance.

**Figure 1:**
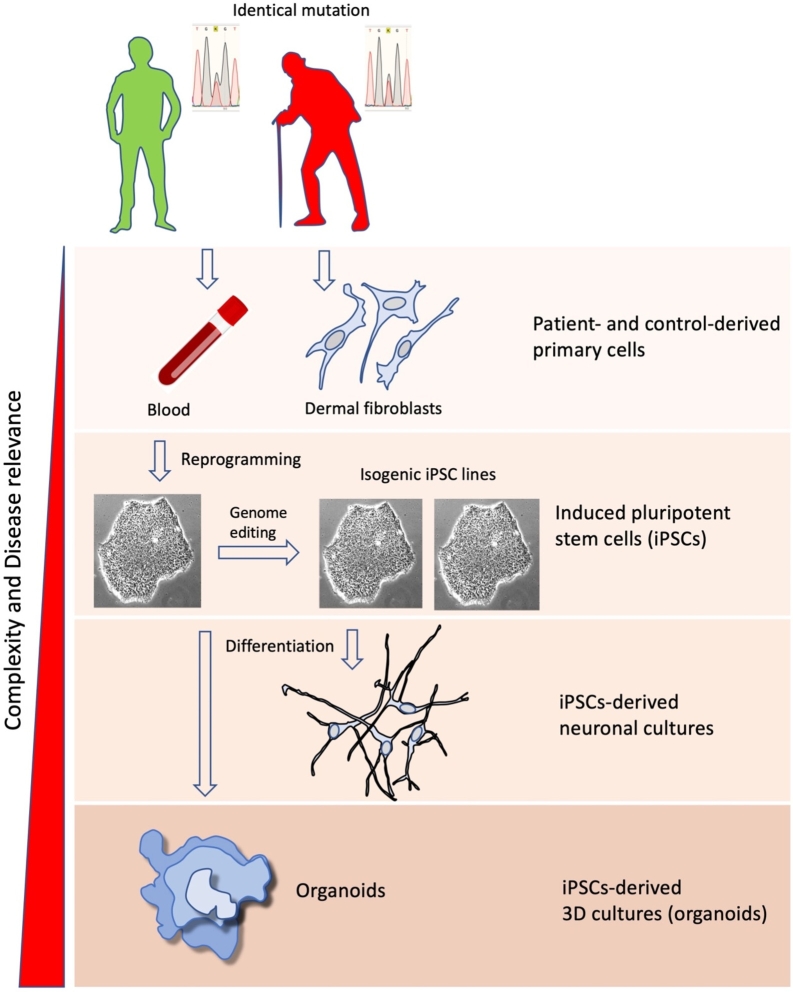
Schematical representation of the steps in developing patient- (and healthy control) derived in vitro models to study reduced penetrance in movement disorders.

Another example is X-linked dystonia-parkinsonism, a neurodegenerative disorder caused by a founder retrotransposon insertion, in which a polymorphic hexanucleotide repeat accounts for ∼50 % of AAO variability [18], [19]. A recent GWAS has identified additional factors modifying AAO [20]. The detected regions harbor or lie adjacent to *MSH3* and *PMS2*, the genes that were recently implicated in modifying AAO in Huntington’s disease [21]. This work indicated the existence of three modifiers of AAO in X-linked dystonia-parkinsonism that likely affect the DNA mismatch repair pathway.

## Techniques to design human cellular models

Insights into disease mechanisms have come mostly from work on patients’ postmortem brain samples, transgenic animals, and various non-neuronal in-vitro models. The development of human induced pluripotent stem cells (iPSCs) [22] has enabled the generation of data from cultures of patient-derived neurons. iPSCs are pluripotent stem cells derived from adult human tissues by introducing four transcription factors (Oct3/4, Sox2, c-Myc, and Klf4), a mechanism named reprogramming. These iPSCs can be differentiated into multiple cell types, creating a model that faithfully recapitulates human genetic context with physiological gene expression levels in the diseased cell type (Figure 1).

This potential is further supported by combining iPSC technology with genome editing. The correction of mutations in patient-derived iPSCs allows to generate isogenic controls and the modification of reporter lines facilitates differentiation towards specific cell types. Genome editing using CRISPR/Cas9 technology enables sequence-specific genome manipulations in cellular models [23]. This method is based on the introduction of targeted double-stranded breaks in DNA through the RNA-guided Cas9 endonuclease. DNA double-strand breaks are typically repaired by one of at least two different pathways: nonhomologous end-joining (NHEJ) and homology-directed repair (HDR). NHEJ results in insertions, deletions, or nucleotide substitutions of various lengths, which can disrupt the translational reading frame of a coding sequence or the binding sites of trans-acting factors in promoters or enhancers. HDR-mediated repair can be applied to introduce specific changes, such as point mutations or to insert desired sequences through recombination of the target locus with exogenously provided DNA templates. Furthermore, a modified version of the technology has been developed to recruit heterologous domains that can regulate endogenous gene expression or label specific genomic loci in living cells [24]. The CRISPR-edited cells differ from their parent line ideally only with respect to the manipulated region, but otherwise retain the full genetic background of the donor.

To further increase the complexity of cellular models, three-dimensional cultures have been developed. These organoids are an advancement of traditional two-dimensional culture systems that allow modelling aspects of organ development, regeneration, and pathophysiology. They are composed of multiple cell types that originate from stem cells by means of self-organization. Neurons in “cerebral organoids” showed properties characteristic of neonatal cortical brain tissues and the organoids contained interdependent domains recapitulating various regions of the brain [25]. Similar strategies have been successfully used to build organoids representing regions of the midbrain [26] or the hippocampus [27]. Despite their promise, organoids still require significant development to unfold their full potential for disease modelling. Organoids from iPSCs often retain immature characteristics and a general limitation is the high variability of the phenotypes that organoids can produce. Therefore, brain organoids have been used so far only in a few studies to model diseases.

Apart from that, there are iPSC-related technical challenges that could introduce artefacts during stem cell modelling in reduced penetrance research. These could include (i) rare copy number variants inherited from the parental somatic cell for which the iPSCs need to be characterized, (ii) different differentiation protocols used by different laboratories, (iii) maturation period of derived neurons or organoids, (iv) risk of cellular heterogeneity due to intense culturing effort of several weeks. These can be addressed partially by standardized protocols and high-throughput analysis techniques.

## Endogenous human neuronal disease models of movement disorders

Studies in iPSC-derived dopaminergic neurons from PD patients demonstrated the significance of oxidative stress, mitochondrial dysfunction, diminished protein turnover, and impaired intracellular trafficking [28]. One of these studies identified a time-dependent pathological cascade in iPSC-derived dopaminergic neurons with mutations in *DJ-1* [29]. Mitochondrial oxidant stress was identified as a first cellular phenotype leading to oxidized dopamine accumulation and ultimately resulting in reduced glucocerebrosidase enzymatic activity, lysosomal dysfunction, and alpha-synuclein accumulation. This toxic cascade was observed in human, but not in mouse, PD patient-derived neurons at least in part because of species-specific differences in dopamine metabolism. Early treatment with mitochondrial antioxidants lowered accumulation of oxidized dopamine and rescued lysosomal dysfunction in patient neurons. Very recently, the potential of midbrain organoids for modeling early developmental changes in PD has been investigated. Single-cell RNA-sequencing data showed that LRRK2 p.G2019S alters neurodevelopment and results in an untimely and incomplete differentiation with reduced cellular variability [30].

For *GBA*-linked PD, a pair of monozygotic twins clinically discordant for PD has been examined using iPSCs, both harboring a heterozygous *GBA* mutation [31]. iPSC-derived dopaminergic neurons from both twins had reduced glucocerebrosidase enzymatic activity, elevated alpha-synuclein protein levels, and a reduced capacity to synthesize and release dopamine. Interestingly, the affected twin’s neurons showed lower dopamine level, increased monoamine oxidase B expression, and impaired intrinsic network activity. Overexpression of wild-type *GBA* and treatment with monoamine oxidase B inhibitors normalized alpha-synuclein and dopamine levels. *GBA* mutant neurons have been further investigated in a recent study that aimed to identify genetic variants in multiple large datasets that affect the penetrance and AAO of *GBA*-associated neurodegeneration [32]. Notably, the two main loci identified that influence disease risk in *GBA* carriers are variants in close proximity to SNCA and CTSB (encoding cathepsin B), both implicated in the lysosomal autophagy pathway. To further investigate the CTSB association, iPSC-derived neurons from *GBA* variant carriers were compared to non-carriers. Neurons from *GBA* variant carriers showed a decrease in active cathepsin B protein levels, suggesting a further reduction in lysosomal protease function in these cases.

Dystonia has been examined in human neurons so far only in a small subset of studies. Patient-specific cholinergic motor neurons have been used to model DYT-*TOR1A* through either direct conversion of patients’ skin fibroblasts or differentiation of iPSCs [33]. *TOR1A* mutant neurons showed reduced neurite length and branches, markedly thickened nuclear lamina, disrupted nuclear morphology, and impaired nucleocytoplasmic transport of mRNAs and proteins compared to controls. Interestingly, the nuclear lamina protein LMNB1 was upregulated in *TOR1A* mutant cells and exhibited abnormal subcellular distribution. Downregulation of LMNB1 ameliorated the cellular defects in patient neurons. In a recent study, we evaluated the role of phosphatidic acid phosphatase (PAP) enzymes in *TOR1A* diseases using iPSC-derived dopaminergic neurons from patients, and mouse models of recessive *TOR1A* disease [34]. We found that Lipin PAP enzyme activity is abnormally elevated in human DYT-*TOR1A* patient neurons and in the brains of different *TOR1A* mouse models. Notably, Lipin activity in patient-derived skin fibroblast was not increased compared to controls suggesting a neuron-specific effect.

Mutations in the transcription factor *THAP1* have been linked to a form of autosomal dominant, isolated dystonia with reduced penetrance. We performed transcriptome analysis on cortical neuronal precursors from iPSC lines derived from manifesting and nonmanifesting *THAP1* mutation carriers and control individuals [35]. Our findings indicate that transcriptional alterations during cortical development influence pathogenesis and penetrance and suggest extracellular matrix organization and deoxyribonucleic acid methylation as mediators of disease protection. Another transcriptome study has been performed in DYT-*THAP1* patients’ iPSC-derived neurons and mouse models to examine how *THAP1* mutations lead to gene expression alterations specifically in the brain [36]. Interestingly, many common dysregulated genes were observed by comparing *THAP1* mutant and control samples, that are involved in dystonic syndromes related pathways, such as synaptic transmission, nervous system development, and locomotor behavior.

In summary, these studies show how iPSCs are well suited for generating patient-specific disease models for genetic movement disorders. Further investigations will elucidate more and more mechanisms behind the phenomenon of reduced penetrance.

## Conclusion and outlook

Progress in the understanding of movement disorders has been remarkable, and the research is advancing rapidly on several fronts, however, there is in most cases still no effective treatment. Understanding disease heterogeneity at the patient cellular level will allow the systematic identification of mechanisms and define therapeutic approaches. Stem-cell-based disease modeling remains a new and rapidly developing field that has already demonstrated its ability to deliver a greater understanding of the disease. To exploit these human disease models, the application of high-throughput analysis techniques and large-scale perturbation tools to iPSC-derived neurons and organoids will be required.
